# Alcohol Emergence: A Study on Various Risk Factors and Clinical Characteristics in Patients With Cerebral Venous Sinus Thrombosis From a Tertiary Care Hospital in South India

**DOI:** 10.7759/cureus.65528

**Published:** 2024-07-27

**Authors:** Sivaji Murugaiyan, Akshaya Rathin Sivaji, Asir Julin

**Affiliations:** 1 Neurology, Tamil Nadu Government Multi Super Specialty Hospital, Chennai, IND; 2 Internal Medicine, Panimalar Medical College Hospital & Research Institute, Chennai, IND

**Keywords:** neurological infections, alcohol use, prothrombotic conditions, cerebral venous sinus, cerebral venous sinus thrombosis (cvst)

## Abstract

Objective and aim

Cerebral venous thrombosis (CVT) is a rare condition with significant morbidity and mortality risk involving the thrombosis of cerebral veins and dural sinuses. Its symptoms vary widely, ranging from headaches to focal neurological deficits, posing significant challenges to its diagnosis. Various contributing factors are involved in its etiology, some of which are gender specific. The clinical presentation varies widely and differs based on geographic distribution. This diversity makes CSVT challenging to diagnose, as it manifests in different ways and requires keen medical observation. Early detection is crucial for prognosis, as addressing the underlying cause can prevent further complications. This study aims to identify various causative factors and clinical presentations observed in individuals diagnosed with cerebral venous sinus thrombosis (CVST).

Material and methods

This prospective observational study was conducted at the Department of Neurology for a year, involving 55 patients admitted to the Neurology OPD/ER. Sample selection was done using non-probability convenience sampling. Patients aged 18 years or older presenting with symptoms indicative of CVST, confirmed by neuroimaging, were included in the study. Patients with arterial stroke, space-occupying lesions, and CVST related to pregnancy or puerperium were excluded. A detailed and structured medical history was obtained, and relevant blood investigations were conducted to find the underlying etiology.

Results

The study population had a mean age of 33 ± 2.04 years. The gender distribution is inclined towards 78.1% males due to the exclusion of pregnancy- or puerperium-related CVST to identify other predominant risk factors. 87.2% of the patients presented acutely, primarily with headaches (94.54%), and 67.2% had seizures within a week of admission. Prothrombotic conditions (45.4%) were the prevalent risk factor in the study group. Second, infections accounted for 21.8%. Alcoholism was seen in 52.7% of the study population, but its correlation with CVST remains unclear. The superior sagittal sinus (34.5%) and transverse sinus (20%) were commonly involved in neuroimaging, with multiple sinus involvement observed in some cases.

Conclusion

Cerebral venous sinus thrombosis (CVST) presents with a wide range of symptoms, making diagnosis challenging. However, with modern imaging and lab tests, we can detect and treat it effectively, often with positive outcomes and minimal long-term effects. This study seeks to understand the risk factors associated with CVST, contributing to its diagnosis and management.

## Introduction

Cerebral venous sinus thrombosis (CVST) is a rare type of venous thromboembolism involved in the etiopathogenesis of stroke. Predominately seen in younger and middle-aged individuals, leading to significant morbidity and mortality. The incidence varies across different population groups. In males <18 years of age, the incidence was notably higher, followed by women aged 18 to 29 years [1]. The incidence of CVST in India is 96 cases per 100,000 individuals, with an equal distribution across genders. Previously, increased preponderance was seen among females, but the current data shows an equal incidence across genders [2]. CSVT results from blood clots in cerebral veins, dural sinuses, and, in rare instances, cortical veins. Clinical presentations of CVST are diverse, often with various underlying etiologies, making its diagnosis challenging. The initial instance of CVST was documented in 1825, during an autopsy of a patient experiencing headaches and seizures, which revealed venous thrombosis in both the superior sagittal sinus and lateral sinus [3].

The clinical presentation of CVST is often misinterpreted for arterial stroke due to its nonspecific symptoms and variability among patients. CVST cases are encountered not only by neurologists/neurosurgeons but also by various specialists in acute care settings, such as ophthalmologists, family physicians, emergency medicine specialists, and pediatricians. This paper aims to explore predisposing risk factors for CVST, along with the timing of presentation, common presenting symptoms, pathophysiology, commonly involved dural venous sinuses, and gender-specific differences. There is a significant gender difference in the etiology and presentation of CVST [4]. There are still newer risk factors to be studied following numerous reports of COVID-19 vaccine-induced CVST [5]. This understanding facilitates prompt diagnosis and expedited intervention.

## Materials and methods

This is a prospective observational study conducted at the Department of Neurology. The duration of the study was one year, from April 12th, 2023, to April 17th, 2024. This study included 55 patients who met the inclusion and exclusion criteria during the study period and were admitted to the Neurology OPD/ER. The sample size determination and selection were carried out using the non-probability convenience sampling technique. Ethical approval for the study was granted by the Institutional Human Ethics Committee.

Inclusion criteria

Patients aged >18 years of age with symptoms suggestive of CVST, such as headaches, nausea, focal neurological deficits, blurring of vision, cranial nerve deficits, and seizures, were included in the study. The diagnosis was supported by radio imaging such as magnetic resonance imaging (MRI), magnetic resonance venography (MRV), CT, or CT-angiography.

Exclusion criteria

Patients with inconclusive imaging results, those diagnosed with an arterial stroke, metabolic encephalopathy, space-occupying lesions, and pregnancy/puerperal CVST, were excluded from the study.

Data collection

A structured patient history was obtained, including time of onset of symptoms, neurological examination, demographics, age, gender, comorbidities, and risk factors (alcohol, smoking, illicit drug use). A complete blood profile was done for patients, including a complete blood count, PT (prothrombin time), aPTT, INR, bleeding and clotting profile, and screening for hypercoagulable conditions with homocysteine, protein C and S, factor V Leiden, APLA panel, ANA, antithrombin III, antinuclear antibodies, and antibodies to dsDNA. Informed written consent was obtained in their vernacular language for each patient after clearly explaining the objectives of the study. Neuroimaging was used to confirm the diagnosis.

Statistical analysis

The data for the study were tabulated in an Excel sheet (Redmond, USA). Data involving categorical values were tabulated in percentages and continuous values in mean ± standard deviation. The mean differences were compared using a t-test, a chi-square test, and a Fisher's exact test. A p-value of <0.05 was considered to have statistical significance.

## Results

This study included a total of 55 patients diagnosed with CVST. The mean age of the study population was 33 ± 2.04 and consisted of 43 (78.1%) males and 12 (21.8%) females. The study group exhibits a gender disproportion, with a higher number of males, as pregnancy- and puerperium-related CVST were excluded to focus on other potential causes. Demographics and time of presentation are represented in Table 1. 

**Table 1:** Demographics and time of presentation of the study group

Demographics	No. of Patients n=55 (%)
Mean Age ± SD	33 ± 2.04
Male	43 (78.1)
Female	12 (21.8)
Time upon presentation	
Acute (<48 hrs)	48 (87.2)
Sub-acute (2 to 30 days)	6 (10.9)
Chronic (30 days to 6 months)	1 (1.8)

The majority of patients presented acutely, with only seven patients presenting after 48 hours. Regarding presenting symptoms (Table 2).

**Table 2:** Clinical characteristics of patients with cerebral venous sinus thrombosis

Clinical Symptoms	No. of patients n=55 (%)
Headache	52 (94.5)
Holocranial	34 (61.8)
Regional/localized	16 (29
Thunderclap	2 (3.6)
Nausea & Vomiting	26 (47.2)
Altered Sensorium	13 (23.6)
Seizure	37 (67.2)
Generalized tonic clonic seizure (GTCS)	28 (50.9)
Focal seizure	7 (12.7)
Status epilepticus	2 (3.6)
Aphasia	5 (9)
Fever	4 (7.2)
Hemiparesis	9 (16.3)
Monoparesis	4 (7.2)
Papilledema	25 (45.4)
First degree	19 (34.5)
Second degree	6 (10.9)
Sensory symptoms	14 (25.4)
Paresthesia	7 (12.7)
Benumbed sensation	4 (7.2)
Complete loss of sensation	3 (5.4)
Cranial Nerve Involvement	10 (18.1)
CN VI	5 (9
CN VII	2 (3.6)
CN III + IV + VI	2 (3.6)
CN IX + X + XI	1 (1.8)

Headaches were the most common, with the majority experiencing the holo-cranial subtype. Seizures were the second most reported symptom, and generalized tonic-clonic seizures (GTCS) were the predominant seizure semiology. Other notable presenting symptoms were papilledema and sensory symptoms.

Table 3 shows the various risk factors.

**Table 3:** Risk factors of patients with cerebral venous sinus thrombosis

Risk Factors	No. of patients n=55 (%)
Factor V Leiden mutation	1 (1.8)
Protein C Deficiency	5 (9)
Protein S Deficiency	1 (1.8)
Hyper-homocysteinemia	4 (7.2)
Antiphospholipid Antibody Syndrome	2 (3.6)
Anemia	8 (14.5)
Polycythemia	1 (1.8)
Systemic Lupus Erythematous	3 (5.4)
Inflammatory Bowel Disease (Ulcerative Colitis)	1 (1.8)
Thyroid Disease	5 (9)
Hypothyroidism	1 (1.8)
Hyperthyroidism	4 (7.2)
Infections	12 (21.8)
Central nervous system infection	4 (7.2)
Ear and sinus infection	7 (12.7)
Sepsis	1 (1.8 )
Head Trauma	3 (5.4)
AV Malformations	2 (3.6)
Unknown	7 (12.7)

Summarize the notable risk factors observed in the study group. Prothrombotic conditions were identified as the most prevalent risk factor among 25 patients. Infections accounted for the second most common risk factor, with seven patients with ear and sinus infections and four with CNS infections. One patient had preexisting ulcerative colitis at the time of the diagnosis of CVST. Among females, autoimmune conditions such as systemic lupus erythematosus (SLE), anti-phospholipid antibody syndrome (APLA) syndrome, and hypothyroidism emerged as the predominant risk factors. 

A noteworthy finding in our study was the prevalence of alcohol and substance use among patients (Table 4).

**Table 4:** Substance/drug use in the study group

Type of Substance	No. of Patients n=55 (%)
Alcohol	29 (52.7)
Nicotine dependence with alcohol	12 (21.8)
Chewable tobacco	3 (5.4)
Cannabis	5 (9)
Intravenous illicit drug	2 (3.6)
None	16 (29)

Of these 24 patients, were chronically dependent alcohol users. However, existing literature does not significantly support its association with the development of CVST. Non-contrast CT scans were the primary neuroimaging modality performed upon admission, followed by MRI with MRV conducted for all patients. The radiological findings regarding sinus and venous involvement are summarized in Table 5.

**Table 5:** Radiological findings and dural venous sinuses and veins involved SSG: Superior sagittal sinus, TS: Transverse sinus

Veins/Single Sinus Involvement	No of Patients n=55 (%)
Superior Sagittal Sinus (SSG)	19 (34.5)
Transverse Sinus (TS)	11 (20)
Straight Sinus	4 (7.2)
Sigmoid Sinus	3 (5.4)
Cortical Veins	1 (1.8)
Deep Venous System	2 (3.6)
Two Sinuses Involvement	
SSG+ TS	6 (10.9)
SSG + Straight Sinus	2 (3.6)
Straight Sinus + Sigmoid Sinus	3 (5.4)
Three or more Sinuses Involvement	
SSG + TS + Sigmoid sinus	2 (3.6)
SSG + TS + Vein of Galen	1 (1.8)
SSG + TS + Sigmoid sinus + Straight sinus	1 (1.8)
CT/MRI Findings	
Hemorrhagic venous infarct	41 (74.5)
Non-hemorrhagic infarct	7 (12.7)
Subarachnoid hemorrhage	2 (3.6)
Normal brain parenchyma	5 (9)

The most commonly involved sinuses were the superior sagittal sinus in 19 patients, followed by the transverse sinus in 11 patients. Notably, one patient had involvement of all the major sinuses (Table 6).

**Table 6:** Gender comparison of clinical symptoms and etiology in the study group Values are indicated as n (%). P- values were calculated using ^$^: t-test, ^#^: Chi-square test and ^: Fisher exact test; ** Significant p-value (<0.05) The t-test was used for continuous values and the chi-square test for categorical and discrete values. A Fisher exact test was used for categorical and discrete values with a small sample size.

Gender	Male n=43 (%)	Female n=12 (%)	Statistical value	p-value
Age	33.0233 ± 2.387	32.9167 ± 3.778	0.041^$^	0.26
Clinical presentation				
Headache	42 (97.6)	10 (83.3)	3.7416^#^	0.53
Nausea & Vomiting	21 (48.8)	5 (41.6)	0.1935^#^	0.65
Altered sensorium	11 (25.5)	2 (16.6)	0.4131^#^	0.52
Seizure	33 (76.7)	4 (33.3)	8.0304^#^	0.004**
Aphasia	4 (9.3)	1 (8.3)	0.4131^#^	0.92
Fever	2 (4.6)	2 (16.6)	2.0085^#^	0.15
Hemiparesis	7 (16.2)	2 (16.6)	0.001^#^	0.97
Monoparesis	4 (9.3)	0	0.5644^^^	>0.05
Papilledema	16 (37.2)	9 (75)	5.4041^#^	0.02**
Sensory symptoms	10 (23.2)	4 (33.3)	0.5021^#^	0.47
CN involvement	8 (18.6)	2 (16.6)	0.0237^#^	0.88
Risk Factors				
Factor V Leiden mutation	1 (2.3)	0	1^^^	>0.05
Protein- C deficiency	4 (9.3)	1 (8.3)	0.0107^#^	0.92
Protein- S deficiency	1 (2.3)	0	1^^^	>0.05
Hyper-homocysteinemia	4 (9.3)	0	0.5644^^^	>0.05
APLA Syndrome	0	2 (16.6)	0.0444^^^	<0.05**
Anemia	6 (13.9)	2 (16.6)	0.0556^#^	0.81
Polycythemia	1 (2.3)	0	1^^^	>0.05
SLE	0	3 (25)	0.0084^^^	<0.05**
IBD	1 (2.3)	0	1^^^	0.59
Thyroid Disease	4 (9.3)	1 (8.3)	0.0107^#^	0.92
Infection	10 (23.2)	2 (16.6)	0.2388^#^	0.62
Head Trauma	3 (6.9)	0	1^^^	>0.05
AV Malformations	2 (4.6)	0	1^^^	>0.05
Unknown	6 (13.9)	1 (8.3)	0.2668^#^	0.60
Substance/Drug use				
Alcohol	27 (62.7)	2 (16.6)	8.0075^#^	0.004**
Chewable Tobacco	3 (6.9)	0	1^^^	>0.05
Cannabis	4 (9.3)	1 (8.3)	0.0107^#^	0.92
Intravenous illicit drug	2 (4.6)	0	1^^^	>0.05
None	7 (16.2)	9 (75)	15.6824^#^	0.00007**

The table shows a gender comparison of clinical symptoms and risk factors. Alcohol consumption was higher in males with a significant difference (p=0.004). Seizures were notably higher among males, whereas females predominately had papilledema as presenting symptoms with statistical significance.

## Discussion

The frequency of CVST cases is significantly higher, primarily due to early detection through the widespread availability of neuroimaging and increased clinical awareness among physicians. CVST, with its wide array of causes and numerous presenting symptoms, makes the initial diagnosis challenging. The pathophysiology of CVST remains incompletely understood, with current insights largely based on animal model studies. Primarily attributing the pathological mechanisms to thrombosis of the cerebral venous sinuses and cerebral veins. Thrombosis within the sinuses results in occlusion, leading to a retrograde flow of blood into the veins and capillaries. This disrupts arachnoid granulations, elevating intracranial pressure. Consequently, cerebral edema occurs, precipitating venous hemorrhage, papilledema, and venous hypertension [6]. The physiological alterations following CVST develop gradually over hours or days and may continue to progress for weeks before manifesting as noticeable signs and symptoms. CVST has four distinct clinical syndromes: cavernous sinus syndrome, intracranial hypertension, diffuse encephalopathy, and focal neurological syndrome [7]. The demographics of the study population are represented in Table 1.

The clinical presentation of CVST varies widely, with patients experiencing symptoms influenced by factors such as age, comorbid conditions, location, and extent of the thrombosis. Symptoms may differ depending on the involved dural venous sinuses. Common clinical presentations of CVST are headache, altered consciousness, seizures, focal neurological deficits, papilledema, aphasia, dystonic posture, and coma. Uncommon presentations include recurrent transient ischemic attacks, thunderclap headaches, isolated headaches, tinnitus, and multiple cranial nerve deficits. The clinical characteristics of the study group are given in Table 2. Headache is the most common presenting symptom in patients with CVST [8,9], as observed prominently within our study group (52, 94.5%). Headache emerges due to the stretching of nerve fibers within the vessels and vasa nervorum of the obstructed venous sinus, coupled with elevated intracranial pressure. A holo-cranial headache subtype was observed in 34 (61.8%) patients, which has been reported in previous studies [10,11]. Headaches often localize to the side affected by sinus occlusion, particularly with transverse sinus involvement [12,13]. Seizures emerged as the second most common symptom during or within one week of admission. The symptomatology of seizures in CVST is conflicting across studies, and treatment for CVST-induced seizures remains debatable [14,15]. Studies report that the predominant type of seizure in CVST is either focal seizure [16] or GTCS [17,18]. In our study group, GTCS was the predominant seizure semiology. 

Elevated intracranial pressure (ICP) is common in CVST, interrupting axoplasmic transport to the optic nerve and ultimately resulting in papilledema [19]. Christian et al. reported a 20-30% incidence of papilledema in CVST [20]. In our study, 25 (45.4%) had papilledema. Patients with papilledema have high scores for venous stasis on the susceptibility-weighted imaging (SWI) sequence of MRI, which serves as a neuroimaging surrogate marker for ICP in CVST [21]. Cranial nerve (CN) palsy in CVST is attributed to the extension of thrombosis to venous channels, raised intracranial pressure, or direct pressure exerted by the clot itself [22]. Kuehnen et al. first reported CN palsies in CVST [23]. In our study, 10 (18.1%) had a CN deficit. In the study by Halesh et al. and Stolz et al., neurological focal deficits such as hemiparesis, monoparesis, and CN palsies were reported in patients with CVST >45% in the study groups [24,25]. Table 2 shows the symptoms that were commonly presented in our study group. The risk factors for CVST are linked primarily to prothrombotic conditions, as seen across the literature. Inherited prothrombotic conditions that contribute to CVST include antithrombin deficiency, protein C and S deficiency, factor V Leiden mutation, and hyperhomocysteinemia [26]. The G20210A mutation is a less common but noteworthy risk factor [27]. In our study, the most notable condition was protein C deficiency in five patients, followed by hyperhomocysteinemia in four patients. In the BEAST consortium by Cotlarciuc I et al. reported that various genetic studies discovered genes involved in the clotting cascade present a higher risk for thrombosis in venous circulation compared to arterial, suggesting a greater genetic predisposition for CVST [28]. Table 3 shows the risk factors associated with patients with CVST in the study group.

In terms of acquired prothrombotic conditions, pregnancy and the puerperium are the most common causes among females. To focus on other risks, our study excluded these conditions, as they are widely reported. Pregnancy-related CVST events primarily occur in the third trimester and the first six weeks postpartum (puerperium). Risk factors for CVST in the puerperium include maternal hypertension, cesarean delivery, chorioamnionitis, excessive vomiting, and advanced maternal age [29]. In the study by Jerez-Lienas A et al. mentioned that CVST can be the initial presentation in individuals with antiphospholipid antibody syndrome (APLA) and were predominantly found to have lateral sinus involvement [30]. In our study, two patients had APLA syndrome, primarily with sigmoid sinus involvement. One patient had primary-type APLA, and the other had secondary-type APLA syndrome. In the aPL profile, one patient tested double-positive and the other triple-positive.

A notable risk factor in our study was anemia, which was observed in eight patients. The causal relationship between anemia and CVST is supported only by case reports, case series, and various hypotheses of its relation. A study by Coutinho JM et al. found that anemia is a stronger risk factor for CVST in men than in women [31]. Consistent with this, in our study, six patients with anemia were male (predominantly megaloblastic type due to underlying alcoholism), and two were female. Iron plays a crucial role in regulating thrombopoiesis by inhibiting it. Iron deficiency leads to secondary thrombocytosis, which increases blood viscosity. Additionally, iron-deficient microcytes are less deformable than normocytes, resulting in turbulent blood flow and a higher predisposition to thrombosis [32]. The study by Höfler SF et al. found a high prevalence of thyroid dysfunction in CVST, though the underlying association requires further study [33]. In our study, five patients had thyroid disease, of which four were hyperthyroid and one had hypothyroidism. Evidence suggests that the procoagulant effect observed in hyperthyroidism is mediated through the thyroid hormone receptor beta gene (THRB) and hypofibrinolysis due to it [34].

SLE-related CVST is primarily caused by lupus anticoagulant (LAC) deposition and immune-mediated vasculitis, which inhibit protein C and S-enhancing thrombosis [35]. In the study by Shujun W et al. CVST was identified in 0.48% of IBD patients. Predictive factors included anemia, low albumin levels, and elevated D-dimer. CVST can occur during both the active and remission phases of IBD [36]. In our study, one patient had ulcerative colitis. The incidence of CVST due to infectious etiology has significantly decreased in the antibiotic era. The pathophysiology of septic CVST is likely attributed to infectious vasculitis, with the infection commonly spreading through the facial and pterygoid veins [37]. In our study, infections were the second most common risk factor in 12 patients. In the study by Mukamal KJ et al., light-to-moderate alcohol intake was associated with reduced levels of coagulation factors [38]. However, higher consumption was linked to impaired fibrinolytic potential. In the meta-analysis by Chen M et al., no significant association was seen between alcohol consumption and CVST, but a marginally significant association between alcohol consumption and the risk of VTE in women was observed [39].

In our study, the majority of patients had a history of alcohol use, with some also experiencing substance abuse and dependence. Chronic binge alcohol consumption can lead to CVST due to dehydration, poor nutrition, altered platelet activity, coagulation factor dysfunction, and increased blood viscosity. Alcohol use disorder was observed in 29 (52.7%), of which 24 were chronically dependent. Among these, heavy and binge drinking were present in 19 patients, moderate drinking in seven, and daily small drinking in three patients. The entire data on alcohol and substance use in the study group is given in Table 4. While some studies suggest that mild or moderate drinking may be protective [40], most patients in our study were chronic abusers and dependents. A structured clinical psychiatric interview and the 17-item Hamilton Depression Rating scale were administered to all 29 patients, revealing that eight had mild depression (scores 8-16) and three had moderate depression (scores 17-23).

Imaging plays a pivotal role in diagnosing CVST. All patients in our study underwent a non-contrast CT scan upon admission, as it was a prompt investigation for suspected CVST [41,7]. Subsequently, all patients had an MRI brain scan with MRV. In our study group, 17 had positive findings on the CT scan. Among them, 13 showed the classical cord sign (hyperdense vessel sign indicating cord-like hyper-attenuation within the DVS), and four patients showed sulcal effacement and diffuse parenchymal edema. All patients underwent MRI with MRV, with the majority showing classical findings. The T2 sequence showed a hypointense signal within the DVS, while the MRV revealed an absence of flow within the sinuses using the 2D TOF (time of flight) sequence. In a few patients, contrast-enhanced MRV was used to confirm. However, digital subtraction angiography (DSA) is considered the gold standard for diagnosing CVST [42]. It is particularly useful in patients who have negative CTV and MRV results or those requiring endovascular intervention [7]. Signs and symptoms vary depending on the type of sinus involved. In our study, there were cases with sinus involvement (Table 5). Among patients with two sinus involvements, six had both the SSG and TS affected, while three patients had involvement of the straight sinus and sigmoid sinus. In patients with involvement of more than two sinuses, two patients had the SSG, TS, and sigmoid sinuses affected. VI, VII, and VIII CN palsies are commonly associated with sigmoid sinus involvement [43,44,26]. In our study, five patients had VI CN palsy, of which two had sigmoid sinus involvement. IX, X, and XI CN palsies are typically linked to transverse sinus involvement [43-45]. In our study, one patient exhibited similar CN involvement and had TS, SSG, and a vein of Galen thrombosis. However, it is important to note that cranial nerve palsies can occur with the involvement of any sinus.

Hemiparesis, aphasia, and blurred vision are commonly seen in patients with SSG involvement [43,7]. Out of nine patients with hemiparesis, four had involvements with the SSG. Among the five patients with aphasia, three had involvement in the straight sinus, while one had involvement in the deep venous system. Out of 52 patients with headaches, 27 had involvement with SSG. The sinus involvement in these patients ranged from single to two or three sinus involvements. In our study, the number of patients with SSG involvement was frequently observed. However, we cannot determine the full extent of the symptom association from our data alone. All the patients in our study group, upon diagnosis, were started on low-molecular-weight heparin (LMWH), followed by bridging with direct oral anticoagulants (DOAC). Both LMWH and unfractionated heparin (UH) are commonly used for anticoagulation in CVST, but there is no definitive guideline on which anticoagulant is preferable [46,5]. For the bridging of oral anticoagulants, 49 patients were initiated on warfarin, while six patients with protein C and S deficiency were started on rivaroxaban due to the risk of warfarin-induced skin necrosis in these individuals.

In our analysis of gender comparisons of presenting symptoms and risk factors (Table 6), statistical significance was observed with seizures (p = 0.004) and papilledema (p=0.02) among the presenting symptoms. Among females, risk factors such as APLA syndrome (p=0.05) and SLE (p=<0.05) showed statistical significance. Conversely, in males, alcohol emerged as a significant risk factor with statistical significance (p=0.004). Despite this, we do not POSIT alcohol as a potential etiological factor in the development of CVST, aligning with existing literature. Most of them were chronically dependent or occasional users. Despite autoimmune conditions, infections, and trauma being potential etiologies in the development of CVST, many patients had concurrent alcohol use previously or at present. Notably, no significant associations were found with other substance abuse in our study group. Nevertheless, further research is warranted to determine whether alcohol constitutes a significant risk factor in the pathogenesis of CVST.

In our study, the predominant etiologies were prothrombotic conditions and infections. A significant number of patients were alcoholics, but the association between alcohol consumption and CVST requires further study. Consistent with the literature, headaches were the predominant presenting symptom, followed by seizures. The limitations of our study were that the population was from a single center, and the gender proportion was skewed due to the exclusion of pregnancy and puerperium-related CVST. This may have led to a higher reported number of men with alcohol use. Nevertheless, alcohol use was significantly observed among males, which could be attributed to cultural trends, increased unauthorized sales points, and peer group influence. Despite these limitations, we believe our study provides valuable insights into the etiologies and clinical features of CVST.

## Conclusions

Cerebral venous sinus thrombosis is a treatable and reversible condition that predominantly affects young individuals. Its diverse clinical presentation often leads to underdiagnosis due to nonspecific symptoms. It frequently presents with headaches, seizures, nausea, papilledema, and sensory disturbances. Major risk factors for CVST include prothrombotic conditions, infections, autoimmune disorders, anemia, and occasionally trauma. Given the variability in symptoms, a high level of clinical suspicion is crucial. Diagnosis typically involves CT, MRI/MRV imaging, and relevant laboratory tests, enabling accurate identification and prompt treatment. Early intervention not only improves the prognosis but also helps prevent long-term neurological complications. Further studies are required to identify other etiologies and risk factors to better understand their significance in the development of CVST, as this would substantially improve treating future patients.
